# Supplementary figures and images for: Pentosan polysulfate regulates hepcidin 1-facilitated formation and function of osteoclast derived from canine bone marrow
Source: PLoS One. 2022 Mar 17;17(3):e0265596. doi: 10.1371/journal.pone.0265596 (PMC8929557; doi:10.1371/journal.pone.0265596)

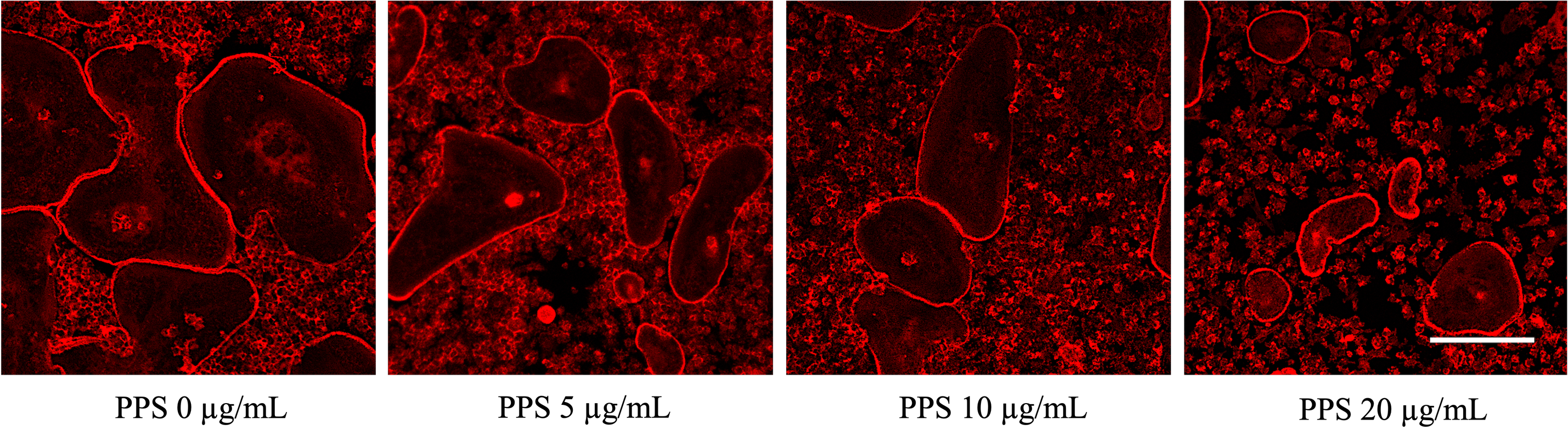

Supplement: S1 Fig — Osteoclasts derived from canine bone marrow were treated with various concentrations (0, 5, 10, 20 μg/mL) of PPS followed by M-CSF (20 ng/mL) and RANKL (50 ng/mL) for 7 days stained with phalloidin, which detects filamentous actin. An actin ring is a characteristic actin structure that is essential for bone resorption by osteoclasts. Scale bar- 100 μm. PPS showed inhibitory effect of formation of number of acting ring and the differention of osteoclast in a concentartion dependent manner. (TIFF) [file pone.0265596.s001.tiff]
