# Supplementary material for: Pentosan polysulfate regulates hepcidin 1-facilitated formation and function of osteoclast derived from canine bone marrow
Source: PLoS One. 2022 Mar 17;17(3):e0265596. doi: 10.1371/journal.pone.0265596 (PMC8929557; doi:10.1371/journal.pone.0265596)
Supplement: S1 Table — (DOCX) [file pone.0265596.s002.docx]

**S1 Table. Primer sequences used to polymerize the osteoclast specific genes**

| **Molecule** | **Sense and anti-sense primer (5-3) ^a^** | **PCR fragment length (base pairs) ^b^** | **Sequence**  **reference** |
| --- | --- | --- | --- |
| **Cathepsin K** | ACCCATATGTGGGACAGGAT  TGGAAAGAGGTCAGGCTTGC | 169 | AY738221 |
| **MMP9** | GGCAAATTCCAGACCTTTGA  TACACGCGAGTGAAGGTGAG | 166 | NM_001003219.2 |
| **NFATc1** | CACAGGCAAGACTGTCTCCA  TCCTCCCAATGTCTGTCTCC | 176 | XM_038653851.1 |
| **FPN1** | CAGTCTATGGGCTGGTGGTG  TCTGGATCGTGATGGCAGTG | 282 | NC_000002.12 |
| **GAPDH** | CTGAACGGGAAGCTCACTGG  CGATGCCTGCTTCACTACCT | 129 | AF327898.1 |

^a^ Primers for forward & reverse sense are presented in a 5ˈ to 3ˈ orientation. ^b^ The expected fragment size. MMP9: [matrix metallopeptidase 9](https://www.ncbi.nlm.nih.gov/gene/403885), NFATc1: [nuclear factor of activated T cells 1](https://www.ncbi.nlm.nih.gov/gene/483925) FPN1: ferroportin 1 and GAPDH: glycer-aldehyde-3-phosphate dehydrogenase.
