# Supplementary material for: Pentosan polysulfate regulates hepcidin 1-facilitated formation and function of osteoclast derived from canine bone marrow
Source: PLoS One. 2022 Mar 17;17(3):e0265596. doi: 10.1371/journal.pone.0265596 (PMC8929557; doi:10.1371/journal.pone.0265596)
Supplement: Attachment — Submitted filename: Response to Reviewers.doc [file pone.0265596.s003.doc]

H.M. Suranji Wijekoon

Graduate School of Veterinary Medicine

Department of Veterinary Clinical Sciences

Laboratory of Veterinary Surgery

Kita 18, Nishi 9, Kita-Ku, (060-0818), Sapporo

Hokkaido, Japan

20.12.2021

Dear Editor-in-Chief,

**Subject: Submission of revised paper**

Thank you for your email dated 1^st^ December 2021 enclosing reviewer’s comments. We have carefully reviewed the comments and have revised the manuscript accordingly. Our responses are given in a point-by-point manner below.

Thank you for your consideration of this manuscript. We hope the revised version is now suitable for publication and look forward to hearing from you in due time regarding our submission and to respond to any further questions and comments you may have. Thank you for your consideration of this manuscript.

[suranjisk@gmail.com](mailto:suranjisk@gmail.com)

Sincerely,

Dr. Suranji Wijekoon (PhD., Mphill., BVSc., MSLCVS)

*(Corresponding Author)*

**Response to reviewer 1:**

Thank you for your review of our paper. We have answered each of your points below.

**[In this manuscript, the authors demonstrate, in vitro, that pentosan polysulfate impairs hepcidin 1-induced osteoclast formation and function. Data is nicely presented and organized]**

Thank you for the valuable comment

**[The animal model choice is not clear. What is the relevance of the canine model for that study? Could not the authors have used PBMC for such purpose? Or rodent bone marrow cells]**

Thank you very much for the comment.

Spontaneous inflammatory arthropathies might be a good model for human juvenile rheumatoid arthritis are available for further clinical perspectives on use of PPS. Enough amount for reliably repeatable use of bone marrow could be obtained from dogs for implication to therapeutic use of PPS to arthritis. It is known that the monocyte/macrophage lineage gives rise to osteoclast. Cells in the bone marrow are mainly precusors that upon maturation migrate out of the bone marrow and enter the circulation, hence it contains high amount of precursor cells.

**[No statistical analysis was reported, yet the authors used asterisks in graphs. Would that indicate statistical significance diferences? Which tests were applied? Gene expression show small error bars, but no asterisks. Please indicate methods used]**

Thank you for the valuable comment.

Data were analyzed using Statistical Package for the Social Sciences v.16 (SPSS inc., Chicago, IL, USA). Statistical significant of quantitative qPCR data, number of OC and resorption pits were determined by analysis of variance (ANOVA), comparing the mean values of the treatments. Where significant different observed, multiple comparison of group means was performed using Post Hoc Bonferroni. The results were considered significant at a 95% confidence level (*p* < .05). All quantitative results are presented as mean ± SE. Fig 4D indicates the gene expression levels of Quantitative qPCR data showed that PPS at 5-10 μg/mL significantly downregulate the gene expression (p<0.05) level of CTK while inhibiting expression level of MMP-9 and NFATc1 in concentration-dependent manner (figure 4D). However, the expression of FPN1 was upregulated toward the higher concentrations of PPS.

**[manuscript present several grammar and typos errors. Please revise]**

Thank you for the valuable comment. All the grammar and typo errors were addressed.

**Response to reviewer 2:**

**[This study is of interest to the field. However, several concerns remain to be resolved, which are listed below.]**

Thank you for the valuable comment

**1. [Hepcidin has been reported to promote the proliferation and differentiation of osteoclast (OC) precursor cells (PMID: 25059214; PMID: 34108442). In addition to inhibiting OC differentiation, does PPS treatment affect the proliferation of OC precursor cells?]**

Thank you very much for your question. We will briefly give the background information of effect of PPS.

We have conducted several studies to understand the PPS activity on OC differentiation, proliferation, and function. All findings were already published in reputed journals. In certain studies, PPS at concentration of 5 μg/mL exerted an inhibitory effect on canine osteoclastogenesis through suppression of key transcription factors such as NFATc1, c-Fos while visualizing co-localization patterns. This information may partially support the suggestion that PPS may exert its inhibitory effect on OC by direct interaction with transcription factors, subsequently deterring the target genes like Cathepsin K and MMP-9 which are needed in bone resorption activity of OC. To further study the effects of PPS on osteoclastogenesis, we examined whether PPS affected RANKL-induced OC function by bone resorption assays and actin formation (Functional structure of OC). The results suggested that PPS at concentrations of 1 and 5 μg/mL suppressed RANKL-induced bone resorption activity and formation of actin-rings of matured OC. The stimulation of M-CSF and RANKL make mature OC result in resorption lacunae, pit formation and actin ring formation which is a prerequisite for OC bone resorption and is the most obvious character of mature OC during osteoclastogenesis. The outcome of this study suggests that the inhibitory action of PPS over OC differentiation and function could be applied in treatment of pathological bone disorders where OC play central role. Intracellular colocalization and interaction of PPS with c-Jun transcriptional factor were observed in this study by immunofluorescence assay emphasizing that the site of action of drug of interest. Binding of c-Fos to the NFATc1 promoter is important for its activation. Suppression of NFATc1 by PPS is the consequence of the downregulation of c-Fos, with the subsequent down-regulation of AP-1 activity and attenuation of OC–specific gene expression required for efficient OC differentiation and bone resorption. Some other finding related to efficacy of PPS over the osteoclast functional unit (actin ring) is added into supplementary files. With these all finding, we concluded that PPS could inhibit the proliferative ability of OC by affecting target genes and functional units.

**2. [Some experiments lack necessary control groups. PPS treatment could inhibit OC differentiation without hepcidin treatment (in Fig 2). What is the underlying mechanism? It is better to include PPS treatments at different doses (e.g., 1, 5, 10 μg/mL) without hepcidin in Fig 3 and Fig 4, so that the effect of PPS on OC differentiation and gene expression with or without hepcidin can be compared.]**

Thank you very much for the comments and valuable suggestions. However, we have already demonstrated the effect of PPS on OC formation and functions. And those were already published. <https://www.ncbi.nlm.nih.gov/pmc/articles/PMC5930774/>. Briefly, we are explaining the underlying mechanism of OC inhibition by PPS. Intracellular colocalization and interaction of PPS with c-Jun transcriptional factor were observed in our previous study by immunofluorescence assay emphasizing that the site of action of drug of interest. Binding of c-Fos to the NFATc1 promoter is important for its activation. Suppression of NFATc1 by PPS is the consequence of the down-regulation of c-Fos, with the subsequent down-regulation of activator protein 1 transcription factor (AP-1) activity and attenuation of OC–specific gene expression required for efficient OC differentiation and bone resorption. Further extension of the study up to detailed work by evaluating specific binding affinity of PPS with specific protein at nuclear, sub nuclear domain or nuclear speckles in OC would be much awarded the PPS as therapeutic perspective.

To avoid the repetition of those findings, we displayed the effect of PPS in different doses (e.g., 1, 5, 10 μg/mL) without hepcidin in fig 2. We have sited previous experiments in this regard to avoid the confusion of readers. And compared the current results with previous findings comparing all most all the aspects. But thank you again for pointing this out.

**3. In Fig 4D, are there any significant differences in the transcription levels of MMP9, NFATc1 and FPN1? It is better to use Q-PCR here, which is more accurate than that of the reverse transcription PCR. In addition, hepcidin is known to regulate the stability of FPN1 protein. How is the transcription level of FPN1 increased after PPS treatment? Does PPS regulate FPN1 expression via a hepcidin-independent manner?**

Thank you for the valuable comments and agreed with your statement.

In figure legend that was mentioned as RT PCR, but it was correctly motioned in material and method. That was corrected and rephased. Thank you. All quantitative results are presented as mean ± SE. Fig 4D indicates the gene expression levels of Quantitative qPCR data showed that PPS at 5-10 μg/mL significantly downregulate the gene expression (p<0.05) level of CTK while inhibiting expression level of MMP-9 and NFATc1 in concentration-dependent manner (figure 4D). However, the expression of FPN1 was upregulated toward the higher concentrations of PPS.

**4. [The published paper and the current manuscript have shown that hepcidin promotes the differentiation of OC precursor cells. In Fig 5, it is better to examine the FPN and iron levels in OC precursor cells. In Fig 5, what are the small size cells surrounding the OCs? If they are the undifferentiated bone marrow mononuclear cell (BMMs), why they did not show reduced FPN levels after hepcidin treatments? The FPN and iron levels were also not changed in these cells after PPS treatments (Fig 5D-G).]**

Thank you for the comment and suggestions for advancement of future experiments.

However, in this study we basically focused on how the PPS act on Hepcidin treated OC at the differentiating and matured stages where they are actively involved in bone resorption in osteoporosis, osteoarthritis, and rheumatoid arthritis. Small cells surrounding the OC are undifferentiated mononuclear cells which remained in the culture slides even after the 7 days. If we compare those mononuclear cells to mature OC, we can’t see very clear FPN changes. But if we carefully see those small cells in the Fig 5.CD, fluorescence intensities are slightly increased combined with PPS treatment. Interesting point in this juncture is selectivity of treatment efficacy of PPS which profusely shows the therapeutic application against OC among other cells. Those mature OC occupy a large area with several nuclei and cells are massive to visualize the changes compared to tiny cells. However, as you suggested it`s a very valuable point to look for the FPN and iron changes quantitatively within OC precursor cells.

**5.[Western blot is a more accurate method to detect the expression level of FPN protein (or MMP9, NFATc1 and so on).]**

Thank you for the suggestion and agreed with it. We definitely include those additional tests to our future experiments to continue this study to further clarify the mode of action of PPS with regards to the iron metabolism in high demanding cells.

**6.[If PPS regulates OC differentiation via changing FPN or iron levels, it would be of interest to add iron mimic such as FAC to see whether it can diminish the effects by PPS.]**

Thank you for the comment.

It very true and we were initially planned to add iron mimic to see how PPS works on it. However, we have already identified and confirmed the efficacy of PPS over the osteoclast genesis and function of OC. Here we more focused on the hepcidin facilitated OC formation and how PPS react over it. This current data very clearly emphasized the ability of PPS on regulating the hepcidin induced OC formation and function. With those fundamentals we will move further to understand the detailed molecular aspects of PPS effect on iron metabolism.

**7. [The OCs numbers in Fig1 and Fig3 do not appear to have significant changes. It would be more convincing to show the entire well instead of the enlarged field of the view.]**

Thank you for the comment and agreed well with it.

We have changed Fig 1 and Fig 3 according to the suggestion. Most profusely Fig 3 was changed adding entire well.

Thank you very much for very constructive and supportive feedback to improve the current layout and to advance the research into the next level in future studies.
